# Early *Plasmodium*‐induced inflammation does not accelerate aging in mice

**DOI:** 10.1111/eva.12718

**Published:** 2018-10-17

**Authors:** Cédric Lippens, Emmanuel Guivier, Sarah E. Reece, Aidan J. O’Donnell, Stéphane Cornet, Bruno Faivre, Gabriele Sorci

**Affiliations:** ^1^ Biogéosciences, CNRS UMR 6282 Université de Bourgogne Franche‐Comté Dijon France; ^2^ Laboratoire IMBE Université Aix Marseille Marseille France; ^3^ Institutes of Evolutionary Biology, and Immunology and Infection Research University of Edinburgh Edinburgh UK; ^4^ IRD UMR CBGP INRA IRD Cirad Montpellier SupAgro Montpellier France

**Keywords:** antagonistic pleiotropy, inflammation, *Plasmodium yoelii*, senescence, survival

## Abstract

Aging is associated with a decline of performance leading to reduced reproductive output and survival. While the antagonistic pleiotropy theory of aging has attracted considerable attention, the molecular/physiological functions underlying the early‐life benefits/late‐life costs paradigm remain elusive. We tested the hypothesis that while early activation of the inflammatory response confers benefits in terms of protection against infection, it also incurs costs in terms of reduced reproductive output at old age and shortened longevity. We infected mice with the malaria parasite *Plasmodium yoelii* and increased the inflammatory response using an anti‐IL‐10 receptor antibody treatment. We quantified the benefits and costs of the inflammatory response during the acute phase of the infection and at old age. In agreement with the antagonistic pleiotropy hypothesis, the inflammatory response provided an early‐life benefit, since infected mice that were treated with anti‐IL‐10 receptor antibodies had reduced parasite density and anemia. However, at old age, mice in all treatment groups had similar levels of C‐reactive protein, reproductive output, survival rate, and lifespan. Overall, our results do not support the hypothesis that the benefits of a robust response to malaria infection in early life incur longer term fitness costs.

## 1 INTRODUCTION

Why organisms’ age has been puzzling evolutionary biologists for decades (Kirkwood & Austad, 2000). Indeed, why should natural selection promote a decline in fitness? The solution to this apparent paradox was provided by Hamilton (1966) who demonstrated that the strength of selection weakens with age. The idea that selection weakens with age is a shared assumption of most evolutionary theories of aging, including the antagonistic pleiotropy hypothesis. Williams (1957) suggested that genes impairing fitness at old age will be selected for if they have fitness‐enhancing roles at young age. Given that the intensity of selection is stronger during early life, the benefit of such pleiotropic genes will outweigh any late‐life cost. The antagonistic pleiotropy hypothesis has attracted considerable attention (Gaillard & Lemaitre, 2017), and indeed, individuals with better performance in early life usually age faster than individuals with poor early performance [see Lemaitre et al. (2015) for a recent review]. The mechanisms underlying these antagonistic pleiotropy effects remain, however, poorly understood. Particularly challenging issues with the study of the antagonistic pleiotropy hypothesis are (a) the identification of genes with antagonistic pleiotropic functions; (b) the assessment of the early‐life fitness benefits; and (c) uncovering the physiological pathways linking early‐life benefits to late‐life costs. An extension of the antagonistic pleiotropy hypothesis, at the phenotypic level, was subsequently proposed (Kirkwood, 1977) and coined the disposable soma hypothesis (Kirkwood & Holliday, 1979). The idea is that energy or resources (sensu* lato*) required to avoid or repair defective cellular machinery (i.e., the maintenance of the somatic line) become unavailable for reproduction, finally resulting in a trade‐off between reproduction and survival.

The inflammatory response is an excellent candidate function with antagonistic properties across lifespan. Inflammation is an essential component of the antimicrobial defense and response to trauma (Medzhitov, 2008). Animal models with knocked‐out genes of the inflammatory response have poor survival prospects during infection [see, for instance, Kurtz, Foreman, Bosio, Anver, and Elkins (2013), and Sorci, Lippens, Léchenault, and Faivre (2017) for a synthesis]. Inflammation is, however, a nonspecific defense that can also damage host tissues, and many age‐associated diseases have an inflammatory origin (including cancer and cardiovascular diseases) (Reverri, Morrissey, Cross, & Steinberg, 2014; Wu, Antony, Meitzler, & Doroshow, 2014). Chronic inflammation is actually a disease status of old age that has been coined “inflammaging” (Candore, Caruso, & Colonna‐Romano, 2010; Vasto et al., 2007). Therefore, while the inflammatory response certainly confers a benefit in terms of immune protection, it can also generate life‐threatening diseases at old age.

In a seminal paper, Finch and Crimmins (2004) analyzed the age‐specific mortality rate of human cohorts in preindustrial Sweden. They showed that high infant mortality (presumably due to infectious diseases in times with rudimental medical care) was associated with high old‐age mortality in the same cohorts. They suggested this “cohort morbidity phenotype” reflects the late negative consequences of chronic inflammation elicited by persistent exposure to pathogenic organisms starting from an early age. However, subsequent studies in other human populations have found no or negative associations, between early‐ and late‐life mortality (Barbi & Vaupel, 2005; Gagnon & Mazan, 2009; Hayward, Rigby, & Lummaa, 2016). Given the correlative nature of historical human data and the potential for confounding effects, it is not surprising that demographic analyses of human cohorts have provided such mixed results (Bengtsson & Broström, 2009; Bengtsson & Lindström, 2003; Catalano & Bruckner, 2006; Myrskylä, 2010).

Experimental approaches are needed to test the role of inflammation as a function with antagonistic properties, potentially accelerating aging (Khan, Agashe, & Rolff, 2017; Pursall & Rolff, 2011). We quantified the early‐life benefits and late‐life costs of the inflammatory response using the malaria parasite *Plasmodium yoelii* and its rodent host. *P. yoelii* induces an inflammatory response that is followed by antibody production (Bakir, Tomiyama, & Abo, 2011; Chen et al., 2010; Couper, Blount, & Riley, 2008; Couper, Blount, Wilson, et al., 2008). We infected young mice and augmented the inflammatory response of one group by administering an anti‐IL‐10 receptor antibody. IL‐10 is one of the principal anti‐inflammatory cytokines that contributes to the regulation of the immune response and the resolution of inflammation (Ouyang, Rutz, Crellin, Valdez, & Hymowitz, 2011). Blocking IL‐10, either at the phenotypic level (with antibodies) or at the genetic level (in knocked‐out models), results in overproduction of pro‐inflammatory mediators and a reduction in parasite density (improved resistance) (Couper, Blount, & Riley, 2008; Redpath, Fonseca, & Perona‐Wright, 2014). We subsequently drug‐cured all mice, after the acute phase of infection, and compared their longevity and reproductive success at old age relative to that of several control groups. If the inflammatory response elicited by *P. yoelii* infection has antagonistic pleiotropic functions, we made two predictions. First, anti‐IL‐10R‐treated mice should better resist the infection than mice only infected with *P. yoelii*. Second, *P. yoelii*‐infected mice (and particularly those also treated with the anti‐IL‐10R antibody) should have lower reproductive output at old age and reduced longevity compared to uninfected control mice.

## 2 MATERIALS AND METHODS

### 2.1 Animals and infections

Sixty female BALB/cJRj mice were purchased (Janvier Labs), housed (six individuals per cage—18.5 × 38 × 22.5 cm—enriched with shelters), maintained under a constant temperature (24°C) and a photoperiod of 12:12 (L:D), and given *ad libitum* filtered water and food (standard mouse pellets).

At 7 weeks of age, mice were allocated to one out of five different groups (*n* = 12 mice per group). Two groups of mice were infected with 10^5^ red blood cells infected with *Plasmodium yoelii* 17XNL by intraperitoneal injection of 100 µl of citrate saline‐diluted blood. One of the groups also received a treatment with an anti‐IL‐10 receptor antibody (intraperitoneal injection of 20 µg of monoclonal anti‐IL‐10R antibodies; 1B1.3a; BD PharMingen). These mice were treated four times, one day prior to *Plasmodium* infection, and at day 1, 3, and 5 postinfection. The dose of anti‐IL‐10R was chosen based on pilot experiments aimed at checking that the treatment did effectively upregulate the inflammatory response without inducing acute lethal immunopathology (Long, Chan, Allen, Read, & Graham, 2008). Control groups of uninfected mice received an intraperitoneal injection with 10^6^ heat‐killed *P. yoelii*‐infected red blood cells, or an intraperitoneal injection of anti‐IL‐10R antibodies at the same dose during four nonconsecutive days, or were left unmanipulated. At day 39 postinfection, all mice were given 7 mg/ml Pyrimethamine (Vetranal^TM^ ‐ Sigma Aldrich) in their drinking water for five consecutive days to clear the infection (Staszewski, Reece, O'Donnell, & Cunningham, 2012).

### 2.2 Early‐life parameters

During the acute phase of the infection, we assessed parasite density at days 3, 5, 7, 10, 12, 14, 17, 20, 24, 27, and 31 postinfection. Parasite density was also assessed at day 55 postinfection to make sure that the antimalarial treatment was effective in clearing the infection. Five microliters of blood were collected from the tail tip, diluted in 200 µl of citrate saline (8.5 g NaCl; 15 g citrate sodium in 1 L of distilled water), and centrifuged at 17,000 *g* for 3 min at 4°C. The supernatant was then removed and the pellet frozen at −80°C. Parasite density was assessed by real‐time PCR on StepOne Real‐Time PCR System of Life Technologies. DNA extraction was performed with the MAGMAX DNA Multi‐Sample Kit (Ambion by Life Technologies) following the protocol provided by the manufacturer with 3 µl of blood diluted in 75 µl of eluent. The amplified gene was *P. yoelii* merozoite surface protein 4/5 gene (PyMSP4/5) using an Applied Biosystems StepOne Plus thermocycler (Applied Biosystems by Life Technologies). A probe was used for fluorescence signal. For each sample and gene, three replicates were carried out in a total volume of 20 µl reaction, which included 12.5 µl TaqMan^®^ Universal PCR Master Mix (Applied Biosystems), 0.75 µl of each primer (10 µM), 2 µl of extracted DNA, 5 µl of probe (10 µM), and 3.5 µl of RNAse/DNase‐free water to complete the total volume. The PCR amplification was as follows: a first step at 50°C for 2 min to activate the probe followed by a denaturation step at 95°C for 10 min, then 40 PCR cycles including denaturation and annealing step at 95°C for 15 s and elongation at 60°C for 1 min. To prove the specificity of the assay, melting curves for all reactions were determined. This procedure consisted of incubations for 15 s at 95°C, 60 s at 60°C, and a final slow heating with a rate of 0.3°C per second up to 95°C with continuous fluorescence measurement. A negative control (water) was added on each plate to ensure the absence of any contamination. A real‐time PCR standard was made using blood from one individual at the peak of the acute phase (day 12 postinfection). A blood smear was made in the morning when parasites are in the ring stage, and parasites counted under optical microscopy (×10,000 red blood cells). We also assessed the number of red blood cells per µl of blood, which allowed us to estimate the number of parasites per µl of blood. This standard blood was serially diluted to have a range of parasite doses and use them as standards in the real‐time PCR. Crossing point values (Cp) were estimated for each sample using the second derivative maximum method implemented in StepOne software (Thermo Fisher scientific, Waltham, MA, USA). Mean Cp values of three replicates were used. The number of parasites per µl of blood was calculated by linear interpolation using the regression between the log_10_ number of parasites per µl in the standard and the associated Cp. The detection threshold was between 5 and 50 parasites per µl of blood. PyMSP4/5 primers used were 5’‐ATCCATCTACTAGTTTGAATCAAAATGA‐3’ for the forward primer and 5’‐CCATTTAGTTTATCTGATTTGTTTGTATTT‐3’ for the reverse primer (94pb). The probe sequence was 5’‐6FAM‐TCATAATTCAAACCCAGATGC‐MGB‐3’.

We counted red and white blood cells at days 0, 5, 7, 10, 12, 14, 17, 20, 24, 27, and 31 postinfection. Two microliters of blood were collected from the tail tip, diluted in 18 µl of PBS, and stored on ice before measurement. Red and white blood cells were counted using a SCIL Vet abc Plus+ hematology analyzer.

At day 3 postinfection, we also assessed three cytokines (IL‐6, IL‐10, IFN‐γ) and one chemokine (CXCL10) as markers of the inflammatory response in the blood. We collected ca. 100 µl of blood from the maxillary vein, using a sterile lancet needle. Blood was immediately centrifuged at 4°C and 4,000 *g* for 10 min. Plasma was immediately stored at −80°C. We used the Luminex technology on a Bio‐plex200 instrument (Biorad®, USA) to assess the concentration of IL‐6, IL‐10, IFN‐ γ, and CXCL10, with a Milliplex® Map kit (MCYTOMAG‐70 K, Millipore, Germany). A total of 25 µl of plasma was used, following the manufacturer's kit instructions.

### 2.3 Late‐life parameters

We assessed C‐reactive protein in plasma of mice at the age of 469 and 795 days, as a marker of chronic inflammation. We collected ca. 100 µl of blood from the maxillary vein, using a sterile lancet needle. Blood was immediately centrifuged at 4°C and 4,000 *g* for 10 min, and plasma immediately stored at −80°C. We used the Mouse C‐Reactive Protein/CRP Quantikine ELISA Kit (MCRP00) of Bio‐Techne® to assess the concentration of CRP. Plasma samples were diluted by 2,000, and the ELISA assay was conducted following the manufacturer's kit instructions. Each sample was run in duplicate, and the mean values were used in the statistical analyses.

When females were 487 days old [far past the age of reproductive peak (Silver, 1995)], they were placed in individual cages with a male (we used a different male for each female) for 10 days. We defined reproductive success as the probability to breed and litter size at birth. When this first reproductive bout was completed, males were replaced in the females’ cages (we kept the same female–male pair) and the procedure repeated for three reproductive bouts. Females were 613 days old at the end of the third reproductive bout.

Mice were monitored every other day to check their health status and survival. Mice that reached the endpoint (Organization for Economic Cooperation & Development, 2000) were humanely euthanized.

### 2.4 Statistical analyses

We used general linear mixed‐effect models to test the effect of experimental treatments on parasite density, red blood cell, and leukocyte counts (log‐transformed) over the course of the acute infection (PROC MIXED, SAS). Models included time postinfection, squared time, and experimental group as fixed factors (plus interactions), and mouse identity as a random factor. Degrees of freedom were approximated using the Satterthwaite method. Although the general linear mixed‐effect model indicated that the two infected groups had different parasite densities (*F*_1,222_ = 9.15, *p* = 0.0028), the model poorly fitted the data and using different distributions of errors or data transformation did not improve the fit. We therefore used a nonparametric test (Wilcoxon two‐sample exact test; PROC NPAR1WAY, SAS) to compare cumulative parasite density per individual (the sum of parasite density up to day 31 p.i.) between the two *P. yoelii*‐infected groups. Two individuals were removed from this analysis because they died during the acute phase of the infection (one in the *P. yoelii* group and one in the *P. yoelii* + anti‐IL‐10R group).

Differences between experimental groups in cytokines in plasma at day 3 postinfection and C‐reactive protein level at the age of 469 and 795 days were analyzed using a Kruskal–Wallis test (PROC NPAR1WAY, SAS).

The probability to breed at old age was analyzed using a generalized linear mixed‐effect model with a binomial distribution of errors (PROC GLIMMIX, SAS). Reproductive bout and experimental group (plus the two‐way interaction) were included as fixed factors and mouse identity as a random factor. Litter size was analyzed using a generalized linear mixed‐effect model with a Poisson distribution of errors (PROC GLIMMIX, SAS). This model included the same fixed and random terms of the previous one. We also tested whether the overall probability to breed and the cumulative litter size over the three reproductive bouts (the sum of litter size per individual) differed among experimental groups, using a Fisher's exact test (PROC FREQ, SAS) and a generalized linear model with a Poisson distribution of errors (PROC GENMOD, SAS), respectively. Overdispersion was corrected in this last model by scaling the deviance (deviance/*df* = 1); using a zero‐inflated Poisson distribution of errors provided the same results. Finally, we used a generalized linear model with a Poisson distribution of errors (PROC GENMOD, SAS), and scaled deviance, to investigate whether cumulative litter size of females that did breed (therefore excluding those with zero cumulative litter size) differed among experimental groups.

Differences in age‐dependent mortality and lifespan among experimental groups were analyzed with a log‐rank test (PROC LIFETEST, SAS) and a one‐way ANOVA (PROC GLM, SAS), respectively.

## 3 RESULTS

### 3.1 Acute infection

**Figure 1:**
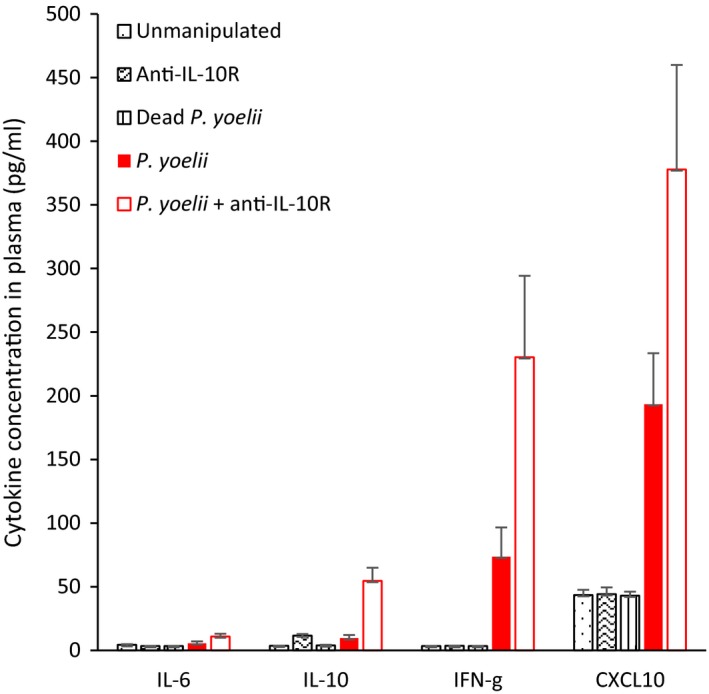
IL‐6, IL‐10, IFN‐γ, CXCL10 concentration (pg/ml) in plasma of mice in the different experimental groups (means ± *SE*)

**Figure 2:**
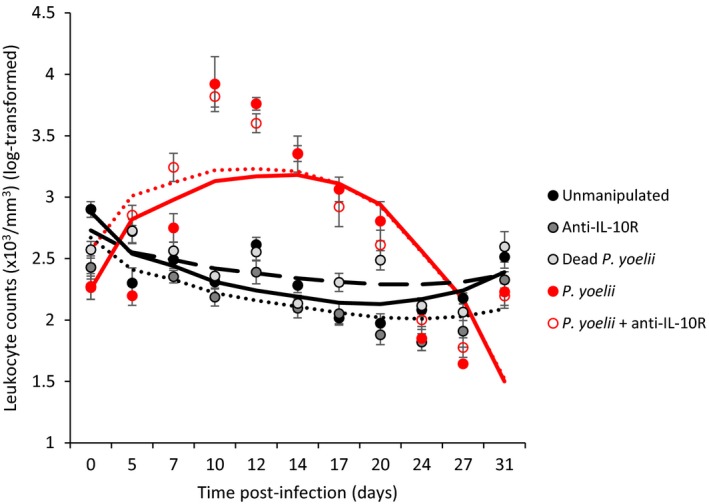
Leukocyte count (×10^3^/mm^3^) in the different experimental groups during the course of the acute infection. Dots represent means (±*SE*) while lines represent model predictions

Infection with *Plasmodium yoelii* is expected to upregulate the inflammatory response during the acute phase. In line with this expectation, we found that the cytokines IL‐6, IL‐10, IFN‐γ, and the chemokine CXCL10 were upregulated in *P. yoelii*‐infected mice, and especially so in infected mice that also received the anti‐IL‐10R treatment (Kruskal–Wallis: IL‐6, $\chi^{2}$ = 27.01, *p* < 0.0001; IL‐10, $\chi^{2}$ = 34.56, *p* < 0.0001; IFN‐γ, $\chi^{2}$ = 33.61, *p* < 0.0001; CXCL10, $\chi^{2}$ = 24.72, *p* < 0.0001; *n* = 55; Figure 1). Similarly, we found that *Plasmodium*‐infected mice had a sharp rise in leukocyte counts at day 10 and 12 p.i. compared to the uninfected groups (Figure 2, Table 1).

**Table 1:** General linear mixed‐effect model exploring the effect of time postinfection, squared time postinfection, and experimental groups on changes in white blood cell count (log‐transformed) during the acute phase of the infection (up to day 31 postinfection)

Sources of variation	*Estimate ± SE*	*F*	*df*	*p*
Time p.i.	−0.041 ± 0.014	5.98	1,575	0.0147
Squared time p.i.	0.001 ± 0.0004	35.88	1,574	<0.0001
Treatment				
Unmanipulated	0.151 ± 0.150	4.90	4,513	0.0007
Anti‐IL−10R	−0.062* *±* *0.150			
Dead * Plasmodium yoelii*	0			
*P. yoelii*	−0.481* *±* *0.151			
*P. yoelii* + anti‐IL‐10R	−0.161* *±* *0.150			
Time p.i. × Treatment				
Unmanipulated	−0.036 ± 0.020	49.68	4,575	<0.0001
Anti‐IL−10R	−0.016* *±* *0.020			
Dead *P. yoelii*	0			
*P. yoelii*	0.182* *±* *0.021			
*P. yoelii* + anti‐IL‐10R	0.153* *±* *0.021			
Squared time p.i. × Treatment				
Unmanipulated	0.001 ± 0.001	63.24	4,574	<0.0001
Anti‐IL−10R	0.0003* *±* *0.001			
Dead *P. yoelii*	0			
*P. yoelii*	−0.006* *±* *0.001			
*P. yoelii* +anti‐IL‐10R	−0.006* *±* *0.001			
Random factor		*z*		
Mouse identity	0.013* *±* *0.006	2.29		0.011

**Figure 3:**
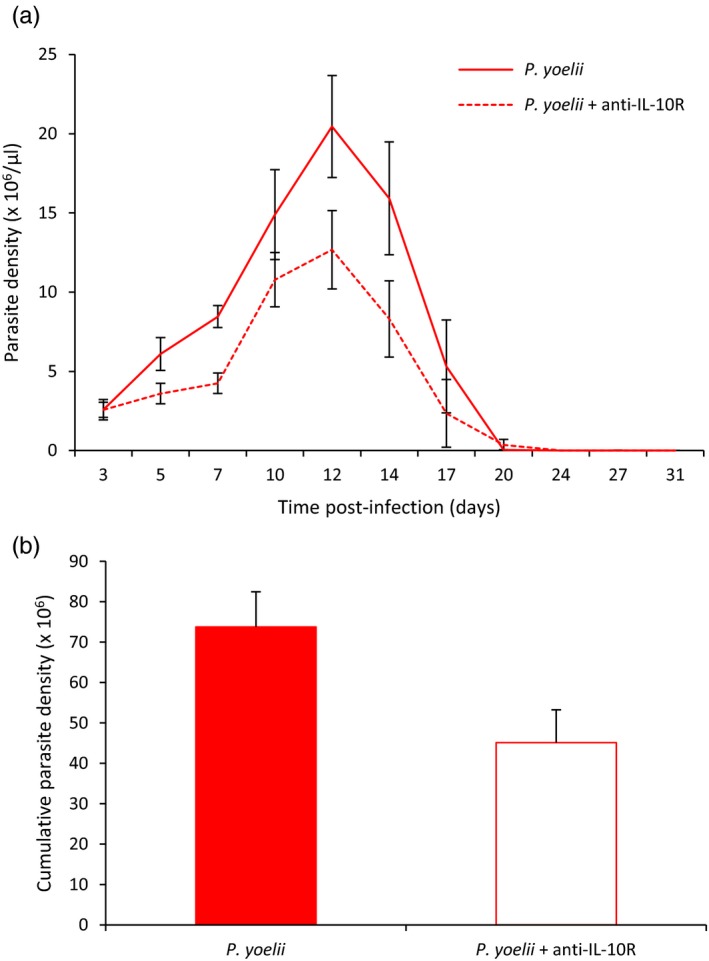
(a) Parasite density (×10^6^/µl) over the course of the acute phase of the infection for the two *Plasmodium yoelii*‐infected groups (means ± *SE*). (b) Cumulative parasite density (sum up to day 31 p.i.) for the two *P. yoelii*‐infected groups (means ± *SE*)

Parasite density peaked at day 12 postinfection for both groups of infected mice (Figure 3a). Cumulative parasite density was lower in the *P. yoelii* + anti‐IL‐10R group (Wilcoxon two‐sample exact test, *p* = 0.0041, Figure 3b), this result being particularly driven by differences at days 5 (Wilcoxon two‐sample exact test, *p* = 0.0423) and 7 postinfection (Wilcoxon two‐sample exact test, *p* = 0.0014). At day 55, after the Pyrimethamine treatment, none of the mice had detectable parasites.

**Figure 4:**
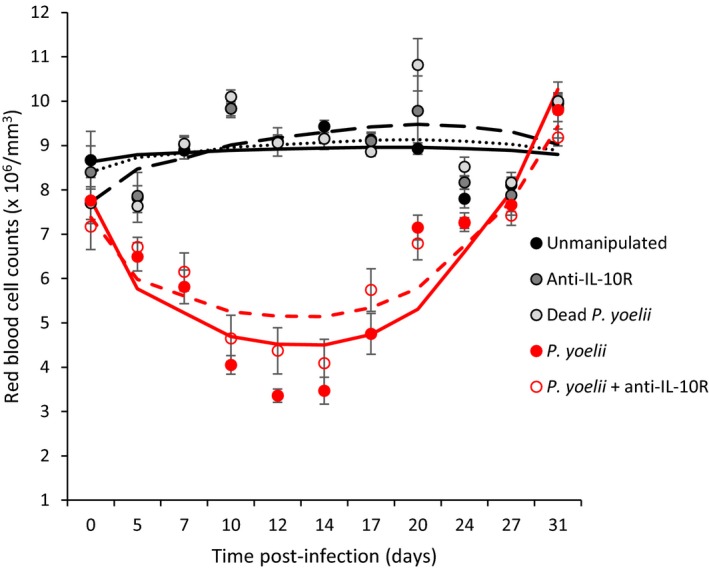
Red blood cell counts (×10^6^/mm^3^) over the course of the acute phase of the infection for the five experimental groups. Dots represent means (±*SE*) while lines represent model predictions

All infected mice became anemic, with minimum red blood cell values reached at day 12 and 14 postinfection (Figure 4, Table 2). However, treating mice with the anti‐IL‐10R antibody reduced anemia of infected mice (Figure 4, Table 3). All infected mice had recovered by day 80 p.i., exhibiting similar red blood cell densities to the uninfected control groups [means ± *SE* (×10^6^/mm^3^), unmanipulated: 8.81 ± 0.16; anti‐IL‐10R: 8.59 ± 0.15; dead *P. yoelii*: 8.75 ± 0.12; *P. yoelii*: 9.08 ± 0.14; *P. yoelii* + anti‐IL‐10R: 9.02 ± 0.25; one‐way ANOVA: *F*_4,53_ = 1.36, *p* = 0.2595].

**Table 2:** General linear mixed‐effect model exploring the effect of time postinfection, squared time postinfection, and experimental groups on changes in red blood cell count during the acute phase of the infection (up to day 31 postinfection)

Sources of variation	*Estimate ± SE*	*F*	*df*	*p*
Time p.i.	0.171 ± 0.045	30.46	1,575	<0.0001
Squared time p.i.	−0.004 ± 0.001	64.54	1,574	<0.0001
Treatment				
Unmanipulated	0.916 ± 0.467	2.48	4,514	0.0432
Anti‐IL‐10R	0.697* *±* *0.469			
Dead * Plasmodium yoelii*	0			
*P. yoelii*	0.061* *±* *0.470			
*P. yoelii* + anti‐IL‐10R	−0.340 ± 0.468			
Time p.i. × Treatment				
Unmanipulated	−0.135 ± 0.063	40.67	4,575	<0.0001
Anti‐IL‐10R	−0.098* *±* *0.063			
Dead *P. yoelii*	0			
*P. yoelii*	−0.664* *±* *0.064			
*P. yoelii* + anti‐IL‐10R	−0.516* *±* *0.064			
Squared time p.i. × Treatment				
Unmanipulated	0.003 ± 0.002	54.16	4,574	<0.0001
Anti‐IL‐10R	0.002* *±* *0.002			
Dead *P. yoelii*	0			
*P. yoelii*	0.023* *±* *0.002			
*P. yoelii* + anti‐IL‐10R	0.017* *±* *0.002			
Random factor		*z*		
Mouse identity	0.126 ± 0.056	2.25		0.0122

**Table 3:** General linear mixed‐effect model exploring the effect of time postinfection, squared time postinfection, and experimental groups on changes in red blood cell count during the acute phase of the infection (up to day 31 postinfection). Only the two infected groups (*Plasmodium yoelii* and *P. yoelii* + anti‐IL‐10R) were considered here

Sources of variation	*Estimate ± SE*	*F*	*df*	*p*
Time p.i.	−0.346 ± 0.047	161.24	1,222	<0.0001
Squared time p.i.	0.013 ± 0.001	246.92	1,220	<0.0001
Treatment				
*P. yoelii*	0.390* *±* *0.520	0.56	1,135	0.4541
*P. yoelii* + anti‐IL‐10R	0			
Time p.i. × Treatment				
*P. yoelii*	−0.150* *±* *0.066	5.12	1,222	0.0247
*P. yoelii* + anti‐IL‐10R	0			
Squared time p.i. × Treatment				
*P. yoelii*	0.005* *±* *0.002	6.82	1,220	0.0096
*P. yoelii* + anti‐IL‐10R	0			
Random factor		*z*		
Mouse identity	0.364 ± 0.170	2.14		0.0160

### 3.2 Carryover effects

We investigated whether early infection and an enhanced inflammatory response had carryover effects in terms of chronic inflammation by assessing CRP in plasma when mice were 469 and 795 days old. Mice in the different experimental groups had similar CRP values, both at the age of 469 days and at the age of 795 days (age = 469 days: Kruskal–Wallis $\chi^{2}$ = 2.08, *p* = 0.721; age = 795 days: Kruskal–Wallis $\chi^{2}$ = 7.26, *p* = 0.123; Supporting Information Appendix S1: Figure S1).

**Figure 5:**
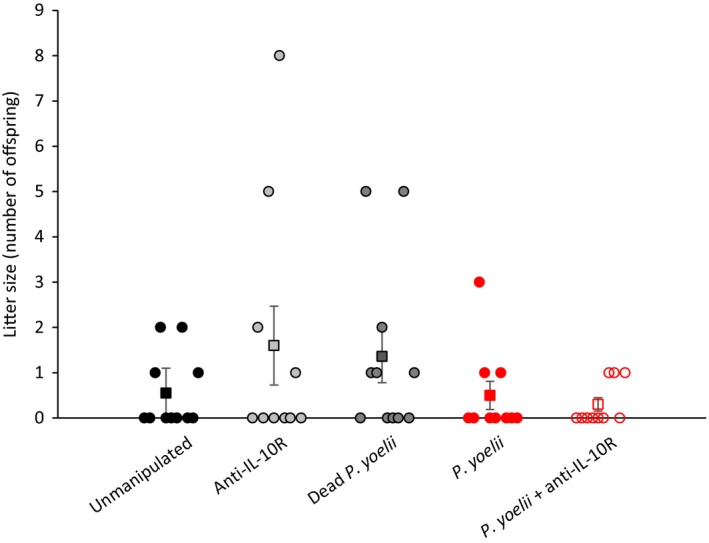
Cumulative reproductive success (sum of litter size at birth over three consecutive reproductive bouts) for the five experimental groups. Each dot represents individual values, while squares represent the mean values (±*SE*) for each experimental group

Reproductive success at old age was assessed as the probability to breed and as litter size over three reproductive bouts. Although many females did not breed at all because of their advanced age, the probability to breed significantly decreased over the three reproductive bouts (Table 4), but the rate of decrease was similar for the different experimental groups (nonsignificant interaction between reproductive bout and treatment, Table 4). When using litter size as a measure of reproductive success, the results were similar: Litter size decreased over time but at a similar rate for the different experimental groups (Supporting Information Appendix S1: Table S1). The comparison of the overall probability to breed and the cumulative litter size (sum of the litter size over the three reproductive bouts) confirmed that there were no differences among the experimental groups (Fisher's exact test, *p* = 0.7928; generalized linear model, $\chi^{2}$ = 7.77, *p* = 0.1003, *n* = 52; Figure 5). Finally, we compared cumulative litter size among experimental groups using only females that did reproduce. Again, this model showed that there were no differences among groups (generalized linear model, $\chi^{2}$ = 7.62, *p* = 0.1067, *n* = 20).

**Table 4:** Generalized linear mixed‐effect model (with a binomial distribution of errors) exploring the effect of reproductive bout and experimental group on the probability to breed

Sources of variation	*Estimate ± SE*	*F*	*df*	*p*
Reproductive bout	−1.139 ± 0.591	4.88	1,99	0.0295
Treatment				
Unmanipulated	−2.137 ± 1.750	1.33	4,99	0.2655
Anti‐IL−10R	−0.478* *±* *1.697			
Dead * Plasmodium yoelii*	0			
*P. yoelii*	0.015* *±* *1.989			
*P. yoelii* + anti‐IL‐10R	−4.591 ± 2.307			
Reproductive bout × Treatment				
Unmanipulated	0.758 ± 0.862	1.20	4,99	0.3160
Anti‐IL−10R	0.124* *±* *0.868			
Dead *P. yoelii*	0			
*P. yoelii*	−0.743* *±* *1.243			
*P. yoelii* + anti‐IL‐10R	1.734* *±* *1.000			
Random factor				
Mouse identity	1.445 ± 0.827			

**Figure 6:**
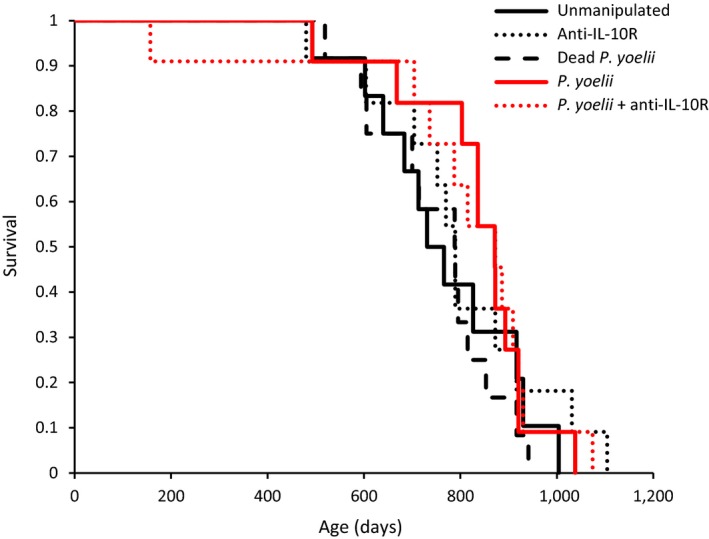
Age‐dependent survival for the five experimental groups

Lifespan did not differ among experimental groups (one‐way ANOVA: *F*_4,51_ = 0.44; *p* = 0.7821). Similarly, the analysis of time‐dependent mortality did not show any difference among groups (log‐rank test: $\chi^{2}$ = 2.98; *p* = 0.5606; Figure 6).

## 4 DISCUSSION

The antagonistic pleiotropy is one of the most studied hypotheses to explain the evolution of aging. However, the vast majority of studies have focused on the trade‐off between early and late expressed fitness traits, neglecting the molecular and/or physiological functions underlying these trade‐offs (Lemaitre et al., 2015). Here, we provide the first experimental test of the antagonistic pleiotropy properties of the inflammatory response in a vertebrate species. Our aim was twofold: i) to assess the early benefits of the inflammatory response during the acute phase of a *Plasmodium* infection and ii) quantify the costs paid at late age. We found that infection does activate the inflammatory response and that mice with the strongest inflammatory response (*P. yoelii* + anti‐IL‐10R group) had lower parasite density. Mice in the *P. yoelii* + anti‐IL‐10R group were also less anemic compared to *P. yoelii*‐infected individuals. These results indicate, therefore, that inflammation in response to *P. yoelii* infection confers benefits. They also corroborate previous findings showing that IL‐10 plays a crucial role in regulating the immune response against *Plasmodium* and therefore in host resistance and pathology (Couper, Blount, & Riley, 2008; Couper, Blount, Wilson, et al., 2008; Freitas do Rosario & Langhorne, 2012). However, we did not find support for the assumption that early‐life inflammation incurs late‐life costs. CRP, late reproductive success, age‐dependent mortality, and longevity were similar among infected and uninfected controls.

Our assessment of the benefits of the inflammatory response was restricted to two proxies of the cost of infection: parasite density and anemia. We used a nonlethal strain (17XNL) of *P. yoelii* because we did not want to induce any mortality during the acute phase of the infection, and therefore, we could not directly compare the survival of mice in the different experimental groups. Nevertheless, there is extensive evidence showing that parasite density and anemia are good predictors of *Plasmodium*‐induced mortality (see, for instance, Laroque et al., 2012; Mackinnon & Read, 2004; Perkins et al., 2011). Keeping parasite proliferation under control and limiting the loss of red blood cells can therefore be seen as good proxies of the likelihood to survive the infection and therefore of early fitness (including reproductive success). Moreover, recent work has shown a direct link between the suppression of pro‐inflammatory cytokines in knockout (KO) mouse models and the probability to die during an infection, due to the substantial increase in parasite burden in KO compared to wild‐type individuals (Sorci et al., 2017).

The idea that early exposure to infectious agents that stimulate the inflammatory response has negative carryover effects at late age rests on the assumption that previously infected individuals have a chronic pro‐inflammatory phenotype, which makes them more vulnerable to a range of diseases. Evidence for such an association between early acute and late chronic inflammation is, however, mixed. For instance, high exposure to microbial infection in a human cohort in the Philippines correlated with lower C‐reactive protein level during adulthood (McDade, Rutherford, Adair, & Kuzawa, 2010), and a Ghanaian cohort living in a malaria endemic area had inherently similar CRP levels to a Dutch cohort (Eriksson, van Bodegom, May, Boef, & Westendorp, 2013). There are also many reports suggesting that early exposure to microbes might have an “educational” role for the immune system by stimulating regulatory mechanisms that prevent autoimmune damage (Rook, 2009). In line with these results, we did not find evidence that *Plasmodium*‐infected mice had upregulated CRP levels at old age.

In addition to having similar CRP levels, mice in the different experimental groups also had similar fitness components, assessed as reproductive success at old age and age‐dependent mortality. Reproductive success was very low for the whole sample of mice [litter size of BALB/c mice at prime age being around 5 pups; Silver (1995)], showing that they were in a status of reproductive senescence. In inbred mouse strains, such as BALB/c, female fecundity is greatly reduced by the age of 10 months (Silver, 1995), and indeed, we found that many females did not breed at all. Nevertheless, the probability to breed and litter size decreased over the three reproductive bouts, albeit the rate of decrease was similar for the different experimental groups. Mean longevity ranged from 752 days for mice that were exposed to dead *P. yoelii* to 831 days for mice that were infected with the parasite, and did not significantly differ among groups. Further, that infected mice lived on average 46 days longer than noninfected mice suggests we did not make a type 2 error due to lack of statistical power. These results, therefore, show that infected mice aged at a similar pace, whatever the treatment they were exposed to when young.

Our study focused on females, and we cannot exclude that males might be more prone to pay late costs of early infection. Sex‐related differences in immune function and parasite resistance have been reported in several studies (see Klein, & Flanagan, 2016 for a review), and therefore, an interesting extension of the current work would be to test for sex‐specific aging in response to early infection and inflammation. Running an experiment with both males and females might further provide the opportunity to explore the role played by other physiological pathways with potentially antagonistic functions through lifespan, such as sexual hormones.

Although the hypothesis that early activation of the inflammatory response might increase late‐occurring mortality was put forward by Finch and Crimmins (2004) based on the analysis of human cohort mortalities, subsequent studies failed to support the hypothesis. In particular, a recent study showed that exposure to infectious diseases in early life does not predict late‐life mortality in a preindustrial population in Finland (Hayward et al., 2016). Our experimental results provide further support to the idea that exposure to infectious diseases in early life does not necessarily accelerate the rate of senescence. However, it should be fully acknowledged that our experimental approach only involved a punctual early infection that was readily cleared by the antimalarial treatment, and animals subsequently faced a pathogen‐free environment. Arguably, this is very different from a natural situation where hosts are repeatedly exposed to infectious threats with a persistent activation of the inflammatory machinery. Therefore, the lack of long‐term fitness cost of early infection might be due to our choice to induce a short, acute, inflammatory response whereas a more persistent chronic inflammatory status might be required to incur late costs. Further work should definitely explore how timing and persistence of an early pro‐inflammatory status affects late‐life fitness traits.

## ETHICAL STATEMENT

All animal experiments were approved by the Comité d'Ethique de l'Expérimentation Animale Grand Campus Dijon, France (CNREEA n C2EA – 105), and by the ‘‘Ministère de la Recherche et de l'Enseignement Supérieur” (project N01867.01) in accordance with the national guidelines (‘“Charte nationale portant sur l”éthique de l'expérimentation animale”, Ministère de la Recherche et de l'Enseignement Supérieur) on the use of animals for research purposes.

## CONFLICT OF INTERESTS

The authors declare no conflict of interests.

## AUTHORS’ CONTRIBUTIONS

G.S and B.F. conceived the study. G.S., B.F. and S.E.R. designed the experiment and obtained funding. A.J.O'D. and S.C. conducted pilot experiments. C.L., E.G., B.F., and G.S. performed experiments. C.L. performed laboratory work. C.L. and G.S. analyzed data. G.S. wrote the first draft of the manuscript. All authors read and approved the final manuscript.

## DATA ACCESSIBILITY

All data have been deposited to DRYAD Digital Repository: https://doi.org/10.5061/dryad.770c174

## References

[eva12718-bib-0001] Bakir, H. Y. , Tomiyama, C. , & Abo, T. (2011). Cytokine profile of murine malaria: Stage‐related production of inflammatory and anti‐inflammatory cytokines. Biomedical Research, 32, 203–208. 10.2220/biomedres.32.203 21673450

[eva12718-bib-0002] Barbi, E. , & Vaupel, J. W. (2005). Comment on “Inflammatory exposure and historical changes in human life‐spans”. Science, 308, 1743. 10.1126/science.1108707 15961654

[eva12718-bib-0003] Bengtsson, T. , & Broström, G. (2009). Do conditions in early life affect old‐age mortality directly and indirectly? Evidence from 19th‐century rural Sweden. Social Science and Medicine, 68, 1583–1590. 10.1016/j.socscimed.2009.02.020 19286293

[eva12718-bib-0004] Bengtsson, T. , & Lindström, M. (2003). Airborne infectious diseases during infancy and mortality in later life in southern Sweden, 1766–1894. International Journal of Epidemiology, 32, 286–294. 10.1093/ije/dyg061 12714551

[eva12718-bib-0005] Candore, G. , Caruso, C. , & Colonna‐Romano, G. (2010). Inflammation, genetic background and longevity. Biogerontology, 11, 565–573. 10.1007/s10522-010-9286-3 20549353

[eva12718-bib-0006] Catalano, R. , & Bruckner, T. (2006). Child mortality and cohort lifespan: A test of diminished entelechy. International Journal of Epidemiology, 35, 1264–1269. 10.1093/ije/dyl108 16723366

[eva12718-bib-0007] Chen, G. , Feng, H. , Liu, J. , Qi, Z. M. , Wu, Y. , Guo, S. Y. , … Cao, Y. M. (2010). Characterization of immune responses to single or mixed infections with *P. yoelii* 17XL and *P. chabaudi* AS in different strains of mice. Parasitology International, 59, 400–406. 10.1016/j.parint.2010.05.005 20609420

[eva12718-bib-0008] Couper, K. N. , Blount, D. G. , & Riley, E. M. (2008). IL‐10: The master regulator of immunity to infection. Journal of Immunology, 180, 5771–5777. 10.4049/jimmunol.180.9.5771 18424693

[eva12718-bib-0009] Couper, K. N. , Blount, D. G. , Wilson, M. S. , Hafalla, J. C. , Belkaid, Y. , Kamanaka, M. , … Riley, E. M. (2008). IL‐10 from CD4+ CD25‐ Foxp3‐ CD127‐ adaptive regulatory T cells modulates parasite clearance and pathology during malaria infection. PLoS Pathogens, 4, e1000004 10.1371/journal.ppat.1000004 18401464 PMC2291447

[eva12718-bib-0010] Eriksson, U. , van Bodegom, D. , May, L. , Boef, A. G. C. , & Westendorp, R. G. J. (2013). Low C‐reactive protein levels in a traditional west‐African population living in a malaria endemic area. PLoS One, 8, e70076 10.1371/journal.pone.0070076 23922912 PMC3724900

[eva12718-bib-0011] Finch, C. E. , & Crimmins, E. M. (2004). Inflammatory exposure and historical changes in human life‐spans. Science, 305, 1736–1739. 10.1126/science.1092556 15375259

[eva12718-bib-0012] Freitas do Rosario, A. P. & Langhorne, J. (2012). T cell‐derived IL‐10 and its impact on the regulation of host responses during malaria. International Journal for Parasitology, 42, 549–555. 10.1016/j.ijpara.2012.03.010 22549022

[eva12718-bib-0013] Gagnon, A. , & Mazan, R. (2009). Does exposure to infectious diseases in infancy affect old‐age mortality? Evidence from a pre‐industrial population. Social Science & Medicine, 68, 1609–1616. 10.1016/j.socscimed.2009.02.008 19269727

[eva12718-bib-0014] Gaillard, J. M. , & Lemaitre, J. F. (2017). The William’s legacy: A critical reappraisal of his nine predictions about the evolution of senescence. Evolution, 71, 2768–2785. 10.1111/evo.13379 29053173

[eva12718-bib-0015] Hamilton, W. D. (1966). The moulding of senescence by natural selection. Journal of Theoretical Biology, 12, 12–45. 10.1016/0022-5193(66)90184-6 6015424

[eva12718-bib-0016] Hayward, A. D. , Rigby, F. L. , & Lummaa, V. (2016). Early‐life disease exposure and associations with adult survival, cause of death, and reproductive success in preindustrial humans. Proceedings of the National Academy of Sciences USA, 113, 8951–8956. 10.1073/pnas.1519820113 PMC4987806 27457937

[eva12718-bib-0017] Khan, I. , Agashe, D. , & Rolff, J. (2017). Early‐life inflammation, immune response and ageing. Proceedings of the Royal Society B, 284, 20170125. 28275145 10.1098/rspb.2017.0125 PMC5360934

[eva12718-bib-0018] Kirkwood, T. B. L. (1977). Evolution of ageing. Nature, 270, 301–304. 10.1038/270301a0 593350

[eva12718-bib-0019] Kirkwood, T. B. L. , & Austad, S. N. (2000). Why do we age? Nature, 408, 233–238. 10.1038/35041682 11089980

[eva12718-bib-0020] Kirkwood, T. B. L. , & Holliday, R. (1979). The evolution of ageing and longevity. Proceedings of the Royal Society B, 205, 531–546. 10.1098/rspb.1979.0083 42059

[eva12718-bib-0021] Klein, S. L. , & Flanagan, K. L. (2016). Sex differences in immune responses. Nature Reviews Immunology, 16, 626–638. 10.1038/nri.2016.90 27546235

[eva12718-bib-0022] Kurtz, S. L. , Foreman, O. , Bosio, C. M. , Anver, M. R. , & Elkins, K. L. (2013). Interleukin‐6 is essential for primary resistance to *Francisella tularensis* live vaccine strain infection. Infection & Immunity, 81, 585–597. 10.1128/IAI.01249-12 23230288 PMC3553820

[eva12718-bib-0023] Laroque, A. , Min‐Oo, G. , Tam, M. , Radovanovic, I. , Stevenson, M. M. , & Gros, P. (2012). Genetic control of susceptibility to infection with *Plasmodium chabaudi chabaudi* AS in inbred mouse strains. Genes and Immunity, 13, 155–163. 10.1038/gene.2011.67 21975430 PMC4912355

[eva12718-bib-0024] Lemaitre, J. F. , Berger, V. , Bonenfant, C. , Douhard, M. , Gamelon, M. , Plard, F. , & Gaillard, J. M. (2015). Early‐late life trade‐offs and the evolution of ageing in the wild. Proceedings of the Royal Society B, 282, 20150209 10.1098/rspb.2015.0209 25833848 PMC4426628

[eva12718-bib-0025] Long, G. H. , Chan, B. H. K. , Allen, J. E. , Read, A. F. , & Graham, A. L. (2008). Experimental manipulation of immune‐mediated disease and its fitness costs for rodent malaria parasites. BMC Evolutionary Biology, 8, 128 10.1186/1471-2148-8-128 18447949 PMC2391164

[eva12718-bib-0026] Mackinnon, M. J. , & Read, A. F. (2004). Virulence in malaria: An evolutionary viewpoint. Philosophical Transactions of the Royal Society B, 359, 965–986. 10.1098/rstb.2003.1414 PMC1693375 15306410

[eva12718-bib-0027] McDade, T. W. , Rutherford, J. , Adair, L. , & Kuzawa, C. W. (2010). Early origins of inflammation: Microbial exposures in infancy predict lower levels of C‐reactive protein in adulthood. Proceedings of the Royal Society B, 277, 1129–1137. 10.1098/rspb.2009.1795 20007176 PMC2842762

[eva12718-bib-0028] Medzhitov, R. (2008). Origin and physiological roles of inflammation. Nature, 454, 428–435. 10.1038/nature07201 18650913

[eva12718-bib-0029] Myrskylä, M. (2010). The effects of shocks in early life mortality on later life expectancy and mortality compression: A cohort analysis. Demographic Research, 22, 289–320. 10.4054/DemRes.2010.22.12

[eva12718-bib-0030] Organization for Economic Cooperation and Development (2000). Guidance document on the recognition, assessment, and use of clinical signs as humane endpoints for experimental animals used in safety evaluation. Environmental health and safety publication series on testing and assessment no. 19. Retrieved from https://ntp.niehs.nih.gov/iccvam/suppdocs/feddocs/oecd/oecd_gd19.pdf

[eva12718-bib-0031] Ouyang, W. J. , Rutz, S. , Crellin, N. K. , Valdez, P. A. , & Hymowitz, S. G. (2011). Regulation and functions of the IL‐10 family of cytokines in inflammation and disease. Annual Review of Immunology, 29, 71–109. 10.1146/annurev-immunol-031210-101312 21166540

[eva12718-bib-0032] Perkins, D. J. , Were, T. , Davenport, G. C. , Kempaiah, P. , Hittner, J. B. , & Ongecha, J. M. (2011). Severe malarial anemia: Innate immunity and pathogenesis. International Journal of Biological Sciences, 7, 1427–1442. 10.7150/ijbs.7.1427 22110393 PMC3221949

[eva12718-bib-0033] Pursall, E. R. , & Rolff, J. (2011). Immune responses accelerate ageing: Proof‐of‐principle in an insect model. PLoS One, 6, e19972 10.1371/journal.pone.0019972 21625631 PMC3097213

[eva12718-bib-0034] Redpath, S. A. , Fonseca, N. M. , & Perona‐Wright, G. (2014). Protection and pathology during parasite infection: IL‐10 strikes the balance. Parasite Immunology, 36, 233–252. 10.1111/pim.12113 24666543

[eva12718-bib-0035] Reverri, E. J. , Morrissey, B. M. , Cross, C. E. , & Steinberg, F. M. (2014). Inflammation, oxidative stress, and cardiovascular disease risk factors in adults with cystic fibrosis. Free Radical Biology & Medicine, 76, 261–277. 10.1016/j.freeradbiomed.2014.08.005 25172163

[eva12718-bib-0036] Rook, G. A. W. (2009). Review series on helminths, immune modulation and the hygiene hypothesis: The broader implications of the hygiene hypothesis. Immunology, 126, 3–11. 10.1111/j.1365-2567.2008.03007.x 19120493 PMC2632706

[eva12718-bib-0037] Silver, L. M. (1995). Mouse genetics. Oxford: Oxford University Press.

[eva12718-bib-0038] Sorci, G. , Lippens, C. , Léchenault, C. , & Faivre, B. (2017). Benefits of immune protection versus immunopathology costs: A synthesis from cytokine KO models. Infection, Genetics and Evolution, 54, 491–495. 10.1016/j.meegid.2017.08.014 28818622

[eva12718-bib-0039] Staszewski, V. , Reece, S. E. , O'Donnell, A. J. , & Cunningham, E. J. A. (2012). Drug treatment of malaria infections can reduce levels of protection transferred to offspring via maternal immunity. Proceedings of the Royal Society B, 279, 2487–2496. 10.1098/rspb.2011.1563 22357264 PMC3350664

[eva12718-bib-0040] Vasto, S. , Candore, G. , Balistreri, C. R. , Caruso, M. , Colonna‐Romano, G. , Grimaldi, M. P. , … Caruso, C. (2007). Inflammatory networks in ageing, age‐related diseases and longevity. Mechanisms of Ageing and Development, 128, 83–91. 10.1016/j.mad.2006.11.015 17118425

[eva12718-bib-0041] Williams, G. C. (1957). Pleiotropy, natural selection and the evolution of senescence. Evolution, 11, 398–411. 10.1111/j.1558-5646.1957.tb02911.x

[eva12718-bib-0042] Wu, Y. Z. , Antony, S. , Meitzler, J. L. , & Doroshow, J. H. (2014). Molecular mechanisms underlying chronic inflammation‐associated cancers. Cancer Letters, 345, 164–173. 10.1016/j.canlet.2013.08.014 23988267 PMC3935998

