# Supplementary material for: Early Plasmodium‐induced inflammation does not accelerate aging in mice
Source: Evol Appl. 2018 Oct 17;12(2):314–23. doi: 10.1111/eva.12718 (PMC6346666; doi:10.1111/eva.12718)
Supplement: Supplementary file 1 [file EVA-12-314-s001.pdf]

Table S1. Generalized linear mixed-effect model (with a Poisson distribution of errors) exploring the effect of reproductive bout and experimental group on litter size at birth.

**Sources of variation (full model)**		*Estimate ± SE*	*F*	*df*	*p*
Reproductive bout		-0.767 ± 0.363	2.91	1,99	0.0913
Treatment	un-manipulated<br>anti-IL-10R<br>dead *P. yoelii*<br>*P. yoelii*<br>*P. yoelii* + anti-IL-10R	-1.629 ± 1.292<br>-1.001 ± 1.033<br>0<br>0.367 ± 1.489<br>-3.772 ± 2.020	1.29	4,99	0.2780
Reproductive bout x Treatment	un-manipulated<br>anti-IL-10R<br>dead *P. yoelii*<br>*P. yoelii*<br>*P. yoelii* + anti-IL-10R	0.514 ± 0.624<br>0.578 ± 0.477<br>0<br>-0.957 ± 1.036<br>1.289 ± 0.838	1.23	4,99	0.3027
**Random factor**					
Mouse identity		1.153 ± 0.471			

**Sources of variation (model without the interaction)**		*Estimate ± SE*	*F*	*df*	*p*
Reproductive bout		-0.448 ± 0.192	5.45	1,103	0.0215
Treatment	un-manipulated<br>anti-IL-10R<br>dead *P. yoelii*<br>*P. yoelii*<br>*P. yoelii* + anti-IL-10R	-0.767 ± 0.701<br>-0.020 ± 0.649<br>0<br>-0.919 ± 0.744<br>-1.292 ± 0.814	1.09	4,103	0.3676
**Random factor**					
Mouse identity		1.153 ± 0.471			

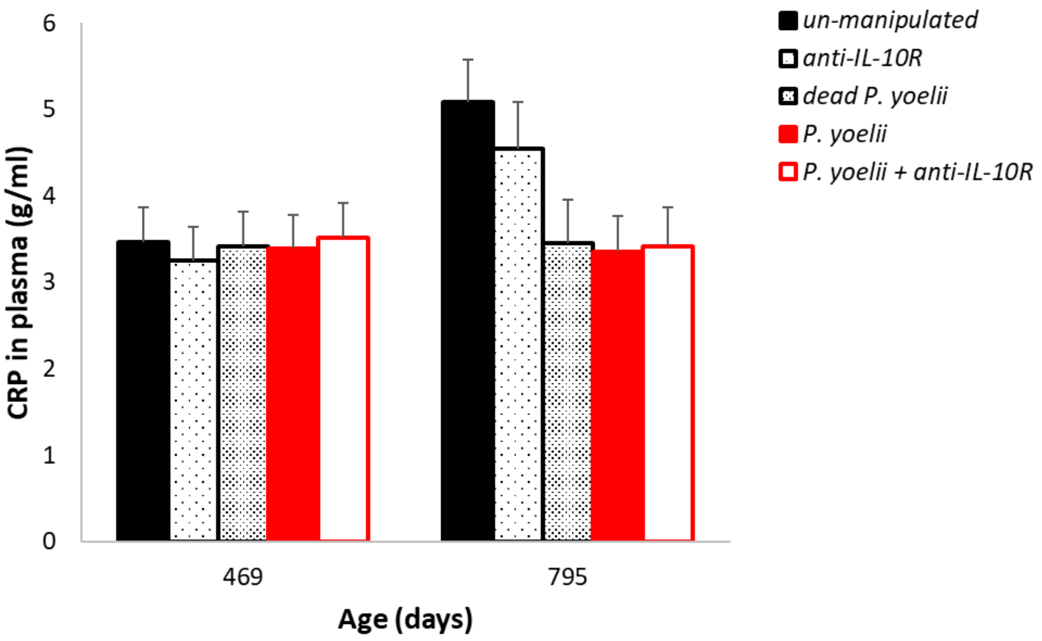

Figure S1. C-reactive protein in plasma for the five experimental groups when mice were 469 and 795 day old (means ± SE).
